# Compatibility of Bioinsecticides with Parasitoids for Enhanced Integrated Pest Management of *Drosophila suzukii* and *Tuta absoluta*

**DOI:** 10.3390/insects15070467

**Published:** 2024-06-22

**Authors:** Fabrizio Lisi, Carmelo Cavallaro, Maria Flavia Pitruzzello, Judit Arnó, Nicolas Desneux, Peng Han, Xingeng Wang, Lucia Zappalà, Antonio Biondi, Antonio Gugliuzzo

**Affiliations:** 1 Department of Agriculture, Food and Environment, University of Catania, 95124 Catania, Italy; fabrizio.lisi@phd.unict.it (F.L.); carmelo.cavallaro@unict.it (C.C.); flavia.pitruzzello@gmail.com (M.F.P.); lucia.zappala@unict.it (L.Z.); antonio.biondi@unict.it (A.B.); 2 Sustainable Plant Protection Program, Institute of Agrifood Research and Technology (IRTA), 08348 Cabrils, Spain; judit.arno@irta.cat; 3 INRAE, CNRS, UMR ISA, Université Côte d’Azur, 06000 Nice, France; nicolas.desneux@inrae.fr; 4 Institute of Biodiversity, School of Ecology and Environmental Science, Yunnan University, Kunming 650504, China; penghan@ynu.edu.cn; 5 USDA ARS Beneficial Insects Introduction Research Unit, Newark, DE 19713, USA; xingeng.wang@usda.gov

**Keywords:** *Ganaspis* cf. *brasiliensis*, *Ganaspis kimorum*, *Necremnus tutae*, biological control, biopesticides, chlorantraniliprole, cyantraniliprole, invasive species, microbials, sublethal effects

## Abstract

**Simple Summary:**

Management strategies targeting invasive pests of agricultural crops have relied heavily on the application of synthetic insecticides. However, sustainable pest management approaches and tools are being effectively implemented. The use of microbials, botanicals, and other natural-based substances alone or in combination with natural enemies is a promising approach. In this context, in the laboratory, we studied the potential synergistic or antagonistic effects in pest control of various biopesticides with *Ganaspis kimorum* and *Necremnus tutae*, two major parasitoids of the spotted wing Drosophila and the South American tomato pinworm, respectively.

**Abstract:**

*Drosophila suzukii* and *Tuta absoluta* are successful biological invaders of agroecosystems. Their integrated pest management (IPM) programs involve the release and/or conservation of natural enemies. Among these, *Ganaspis kimorum* is a major Asian parasitoid of *D. suzukii* and has been introduced as a classical biological control agent of this pest in Europe and North America, while *Necremnus tutae* is a key fortuitous parasitoid of *T. absoluta* in the Mediterranean region. Bioinsecticides represent key alternatives to chemicals for controlling both pests. This study investigated the potential compatibility of both parasitoids with *Beauveria bassiana*, *Bacillus thuringiensis*, garlic essential oil (EO), and spinosad, in comparison to two synthetic insecticides, cyantraniliprole and chlorantraniliprole. The results showed that combining each of the tested insecticides with *G. kimorum* slightly increased pest mortality compared to the insecticide alone. *Necremnus tutae* had a significant additive effect on host mortality when combined with insecticides. *Beauveria bassiana* and *B. thuringiensis* were most compatible with both parasitoid species. Both garlic EO and chlorantraniliprole impaired the survival of immature *N. tutae* and showed sublethal toxicity on the reproductive and non-reproductive behaviors of *N. tutae*. Spinosad exhibited high acute toxicity on both juvenile and adult parasitoids of both species. Overall, these findings provide useful insights into insecticide selectivity toward two key parasitoids and offer new knowledge on the potential of combining natural enemies and bioinsecticides for optimized IPM.

## 1. Introduction

Biological invasions represent one of the most prominent consequences of globalization as a high number of introduced species have been shown to be harmful to agriculture and native biodiversity in newly invaded regions [1,2,3,4]. Arthropod pests are among the major unintentionally introduced species and their invasion trend is projected to increase over time, especially in Europe, where they cause significant economic losses and ecological disruptions [5]. Although current guidelines promote integrated pest management (IPM) programs that minimize chemical inputs, insecticides represent the main tool approached by farmers to manage invasive pest infestations [6,7,8]. This attitude is largely due to the lack of cost-effective control strategies in the invaded regions [9,10,11] and the effectiveness of chemical insecticides for a quick reduction in pest populations [12,13].

However, the reliance on chemicals comes at a significant cost to the environment and human economy due to pesticide bioaccumulation, slow degradation, and non-target effects [14,15], alongside the low tolerance for pesticide maximum residue limits (MRLs), which strongly affect fruit marketability [10]. Moreover, the limited number of chemical molecules registered for invasive species and their routine applications in the field pose additional challenges related to the development of insecticide resistance and the loss of effectiveness of agrochemicals [16]. In response to these challenges, biopesticides have emerged as eco-friendly and cost-effective tools for suppressing arthropod pests in conventional and organic farms. Indeed, these products, which derive from naturally occurring living organisms, such as plants (namely, plant extracts and essential oils), microbials (i.e., bacteria, viruses, and fungi), and animals (e.g., arthropod toxins), are thought to have a negligible or low risk of resistance phenomena and few MRLs restrictions [17].

The increased interest of the pesticide industry in developing safer and promising alternatives to control key invasive pests has significantly improved the relevance of biopesticides over time [18,19,20]. Notable examples include the spotted wing drosophila, *Drosophila suzukii* (Matsumura) (Diptera: Drosophilidae) [21,22], and the South American tomato pinworm, *Tuta absoluta* (Meyrick) (Lepidoptera: Gelechiidae) [18]. These invasive species threaten the worldwide production of soft-skinned fruits and tomato crops, respectively, due to their high reproductive potential and the cryptic nature of their larvae [6,10,23,24]. In particular, *D. suzukii* oviposits on ripe and healthy fruits [25], while *T. absoluta* mainly infests leaves but also the stems and fruits of tomatoes and other solanaceous crops [13] and was recently confirmed to have an epidemiological role in the spread of a tomato virus [26].

Several bioinsecticides have been tested on *D. suzukii* and *T. absoluta*, with spinosad, azadirachtin, and pyrethrins among the most common active ingredients targeting both insect pests. While commonly used bioinsecticides mainly target *D. suzukii* adults, they can also affect pest immature stages and/or natural enemies because of their broad-spectrum action [10,27,28]. Avermectins, essential oils, and microbial-based biopesticides are additional tools for managing *T. absoluta* in organic contexts [18,29,30,31]. In particular, the use of microbials, such as entomopathogenic bacteria (i.e., *Bacillus thuringiensis* (Bt) var. *kurstaki* and *aizawaii*) and entomopathogenic lepidopteran granuloviruses (i.e., members of the genus *Betabaculovirus*), represents a commonly implemented biological control strategy targeting *T. absoluta* larvae [13,32,33]. However, the combined use of biopesticides with macro-biocontrol agents (including parasitoids) needs to be properly assessed to maximize their biocontrol services and the sustainability of IPM programs. In this context, it is worth noting that the origin of a given agrochemical is not always associated with its toxicological features and safety perception [34,35]. 

Various studies have shown the non-target impact of insecticides with different origins and Mechanisms of Action (MoA) towards key natural enemies of *D. suzukii* [28,36] and *T. absoluta* [37,38,39]. However, the potential compatibility of biopesticides with *Ganaspis kimorum* Buffington (Hymenoptera: Figitidae) and *Necremnus tutae* Ribes & Bernardo (Hymenoptera: Eulophidae), which are among the most important larval parasitoids of *D. suzukii* and *T. absoluta*, respectively, is still poorly investigated. Quarantine studies showed the koinobiont endoparasitoid *G. kimorum*, formerly recognized as *G*. cf. *brasiliensis* (Ihering), as the most promising natural enemy for the classical biological control of *D. suzukii* because of its host-specificity [40,41]. Consequently, field releases of this parasitoid are currently ongoing in Europe and the US in the framework of a classical biological control program [42,43]. *Ganaspis* cf. *brasiliensis* consists of a complex of cryptic species (G1-G5), whose taxonomic identity was recently clarified by Sosa-Calvo et al. [44], who re-classified the G1 lineage of *G.* cf *brasiliensis* as *G. kimorum* sp. nov. Meanwhile, the idiobiont ectoparasitoid *N. tutae* is among the most abundant and widespread parasitoids of *T. absoluta* in Europe and Africa [45]. Native to the Mediterranean region, *N. tutae* is a host-feeding parasitoid of *T. absoluta* larvae causing both reproductive and non-reproductive host mortality [46,47]. These attributes, along with its common occurrence in tomato greenhouses, make this parasitoid one of the most promising biocontrol agents to be integrated into IPM programs [48,49].

This study aimed to investigate the non-target effects of field concentrations of three biopesticides (microbials and botanicals) commonly used in organic and/or conventional agroecosystems affected by *D. suzukii* and *T. absoluta* and that of an experimental formulation of garlic essential oil (EO) towards *G. kimorum* and *N. tutae*. Specifically, we assessed the acute toxicity (lethal effect) on parasitoid adults and the sublethal effects on their reproductive capacity (fertility) when exposed to pesticide-treated and infested blueberries (for *D. suzukii*/*G. kimorum*) or tomato leaves (for *T. absoluta*/*N. tutae*), under laboratory conditions. This allowed the simultaneous testing of the lethal and sublethal effects of insecticide residual contact on parasitoids and the combined effect of the insecticide and parasitoid on host mortality. The information may serve as a basis for the potential integration of biopesticides into current biological control strategies against these invasive pests.

## 2. Materials and Methods

### 2.1. Insects

A colony of *D. suzukii* was initiated from field samplings of wild blackberries (*Rubus* sp.) in eastern Sicily, Italy (37°39′47.39″ N, 14°54′3.06″ E), in 2015. Adult flies were reared in insect cages (32.5 × 32.5 × 32.5 cm) (BugDorm^®^, MegaView, Taichung, Taiwan) under standard laboratory conditions according to Lisi et al. [28] with slight modifications (23 ± 1 °C, 55 ± 10% R.H., 16 h:8 h L:D photoperiod). Plastic *Drosophila* vials were filled with cornmeal artificial diet prepared according to Dalton et al. [50] and then provided to adult flies inside the rearing cage to allow them to oviposit, with a honey–water (1:1) solution provided as supplemental food for the flies. The initial colony of the larval parasitoid *G. kimorum* (formerly G1 *G. brasiliensis* in the previous literature, see Lisi et al. [42]) originated from individuals collected in their native range and emerged from *D. suzukii*-infested fruits [51]. A colony of the parasitoid was provided to us through the Sicilian Phytosanitary Service (Regione Siciliana) in 2021, in the context of a classical biological control program [42]. The parasitoid was reared according to Rossi-Stacconi et al. [52]. Briefly, wasps were maintained at 23 ± 1 °C, 60 ± 10% R.H, and a 16 h:8 h L:D photoperiod in a BugDorm^®^ cage and reared on *D. suzukii* larvae in infested blueberries. Infested fruits bearing parasitized larvae were incubated for 30 ± 2 days and newly emerged parasitoids were collected for further rearing or experiments.

Laboratory colonies of *T. absoluta* and the larval parasitoid *N. tutae* were established with specimens collected from organic tomato greenhouses in Sicily, Italy (36°48′01.8″ N, 14°35′11.0″ E) in 2023 and colonies were maintained at 26 ± 1 °C, 60 ± 10% R.H, and 14:10 L.D. as described by Biondi et al. [37]. Briefly, adult moths were released into oviposition cages (50 × 60 × 60 cm) covered with a fine polyester mesh and maintained on potted tomato plants to allow egg laying. Plants were sprayed with honey–water solution (1:1) to feed the insects. 

For the rearing of *N. tutae*, tomato plants infested by larval instars (L2–L3) of *T. absoluta* were exposed to the parasitoids (ratio: ten larvae for each parasitoid couple) for three days in BugDorm^®^ cages (MegaView, Taichung, Taiwan), supplied with honey–water solution (1:1). Following the exposure, tomato plants with parasitized larvae were transferred to other rearing cages for 15 ± 3 days until the emergence of adult wasps.

### 2.2. Insecticides

Among the tested compounds, three were bioinsecticides commonly used for the conventional and organic management of key pests in agroecosystems affected by *D. suzukii* and *T. absoluta*. In particular, these were bioinsecticides of microbiological origin, i.e., *B. thuringiensis* subsp. *kurstaki* strain EG 2348 (Batkur^®^, CBC Europe S.r.l., Grassobbio (BG), Italy), *Beauveria bassiana* strain ATCC 74040 (Naturalis^®^, CBC Europe S.r.l.), and spinosad (Laser^®^, Corteva Agriscience Italia S.r.l., Cremona (CR), Italy). Moreover, an experimental formulation of one botanical, i.e., a garlic EO nanoemulsion (GEO-NE), was tested at a 90% lethal concentration (LC_90_, corresponding to 0.772% of garlic EO) obtained for *T. absoluta* in Ricupero et al. [29]. The effects of these four bioinsecticides were assessed on both host-parasitoid biological models and compared to a conventional insecticide belonging to the diamide chemical class, i.e., chlorantraniliprole (Altacor^®^, FMC Agro Italia, Bergamo (BG), Italy) against *T. absoluta* and cyantraniliprole (Benevia^®^ 2021, FMC Agro Italia) against *D. suzukii*. Insecticides were tested at the recommended field doses (*B. thuringiensis*: 200 mL/hL; *B. bassiana*: 100 mL/hL; spinosad: 25 mL/hL; chlorantraniliprole 12 g/hL; cyantraniliprole: 75 mL/hL) and dilutions were performed individually for each treatment using distilled water.

### 2.3. Lethal and Sublethal Effects of Insecticides on Ganaspis kimorum

*Drosophila suzukii* adults were provided with fresh, untreated, and healthy blueberries for 6–8 h to allow them to oviposit (as described above for fly rearing). Then, infested berries were checked under the microscope to select fruits bearing 8 ± 2 eggs. The day after (i.e., following the egg-hatching), fifteen infested fruits were dipped for ten seconds in each insecticide solution and distilled water was used as an untreated control. Then, the fruits were left to dry for 30 min inside a laminar flow hood. For each treatment and replicate, one treated blueberry was offered to a pair of *G. kimorum* (two- to four-day-old males and females) for 72 h inside an experimental arena (Ø × h: 100 × 50 mm) covered with a mesh net lid to ensure proper ventilation and provided with a foam rubber plug soaked in a honey–water solution (1:1) to feed wasps. Two- to four-day-old parasitoid couples were tested at that age because of their reproductive traits. *Ganaspis kimorum* is a weakly pro-ovigenic species and reaches a peak of mature egg load three to five days after emergence [53]. In addition, parasitoid couples were kept continuously in the experimental arena together with host larvae for 72 h (becoming five to seven days old at the end of the experiment), allowing them to successfully parasitize their respective host larvae. Overall, 15 parasitoid couples (i.e., 15 males and 15 females) were tested per treatment. During the exposure time, parasitoid survival was checked at 24, 48, and 72 h. Afterward, surviving wasps were individually transferred to similar experimental arenas and provided with a new healthy untreated blueberry and a honey–water solution twice a week. *Ganaspis kimorum* mortality was monitored daily for 30 consecutive days or until the wasps died following insecticide exposure. This exposure sequence was kept constant and was chosen to reflect a real field scenario where farmers promptly spray insecticides when *D. suzukii* infestations occur in crops. Notably, this is the same time frame when the inoculative releases of *G. kimorum* are performed in the field in the framework of the classical biological control program.

A complementary bioassay consisted of insecticide- and distilled water-treated fruits, without exposure to parasitoids to evaluate the mortality of *D. suzukii* larvae induced by each tested insecticide. Blueberries of both bioassays (i.e., in the presence and absence of parasitism) were incubated for 30 ± 2 days at 23 ± 1 °C, 60 ± 10% R.H, and 16h:8h L:D photoperiod, and the number of emerged flies and/or parasitoids was counted to assess the *G. kimorum* apparent parasitism (AP), according to Seehausen et al. [40]. The AP was calculated as the proportion of emerged adult parasitoids among the total number of adult insects that emerged from blueberries [40]. Both the individual and combined effects of insecticide and/or parasitoid on pest mortality were assessed.

### 2.4. Lethal and Sublethal Effects of Insecticides on Necremnus tutae

For each treatment and replicate, ten second-instar *T. absoluta* larvae were gently transferred with a soft brush onto a compound tomato leaf consisting of at least five leaflets. The leaf petiole was previously dipped in a vial filled with an agar–water solution at 1% to ensure leaf turgor during the experiments. After all *T. absoluta* larvae had produced mines in the leaf mesophyll, each infested leaf was dipped into the insecticide solution or distilled water for ten seconds and then left to dry for half an hour inside a laminar flow hood. Each treated leaf was then exposed to a pair of *N. tutae* (two- to four-day-old males and females) for 72 h inside an arena (length of 130 mm, width of 80 mm, and height of 70 mm) covered with a net lid where the internal surface was sprayed with a honey–water solution (1:1) to provide food for the wasps during the bioassay. The decision to test two- to four-day-old *N. tutae* pairs was related to the parasitoid reproductive traits. Indeed, this parasitoid species exhibits higher fecundity in its early adult life reaching higher daily mean oviposition rates when five to seven days old [46]. In addition, parasitoid couples were kept continuously in the experimental arena together with *T. absoluta* larvae for 72 h (becoming five to seven days old at the end of the experiment), allowing them to successfully parasitize their respective host larvae. Overall, 15 *N. tutae* couples (i.e., 15 females and 15 males) were tested per treatment.

The mortality of the parasitoids was checked at 24, 48, and 72 h. To determine the relative importance of non-reproductive and reproductive effects of *N. tutae* on its host larvae [47], exposed *T. absoluta* larvae were observed under the microscope at the end of the 72 h of exposure to assess the insecticide effect on the parasitoid–host interactions, i.e., the percentages of (i) parasitized host larvae (parasitism); (ii) killed host through host-feeding (i.e., parasitoid feeding on host hemolymph to continue egg production and maturation) and host-killing (i.e., parasitoid lethally stings host larvae without reproductive output), which are both referred as non-reproductive effects. At the end of 72 h of exposure, surviving wasps were individually moved into similar experimental arenas and provided healthy untreated tomato leaves and a honey–water solution twice a week. The wasps were checked daily for 30 consecutive days or until they died to assess the survival time following the exposure to insecticide residues. In order to evaluate the mortality of *T. absoluta* larvae induced by each insecticide and distilled water in the absence of parasitism, a complementary bioassay was performed without exposing *T. absoluta*-infested leaves to parasitoids. 

Tomato leaves from both bioassays (i.e., in the presence and absence of parasitism) were incubated in aerated arenas (same as above) for 15 ± 3 days at 26 ± 1 °C, 60 ± 10% R.H, and 14:10 L.D., to count the number of emerged pest and/or parasitoid adults and evaluate the individual or combined effect of insecticides and parasitoid on pest mortality. The parasitoid juvenile survival was also assessed as the proportion of *N. tutae* offspring that developed from egg to adult on parasitized host larvae [37].

### 2.5. Data Analysis

Differences in *G. kimorum* and *N. tutae* survival after 24, 48, and 72 h of contact exposure to insecticide residue and differences in pest mortality at the end of the experiment for each tested insecticide were analyzed using the non-parametric Kruskal–Wallis test followed by Dunn’s post-hoc test with Bonferroni-corrected *p*-values (*p* < 0.05) because mortality data did not fulfill the assumptions for the analysis of variance (ANOVA). For each insecticide treatment and tested parasitoid species, differences among treatments in male and female mortality at 24, 48, and 72 h of exposure were analyzed with the Mann–Whitney U test (*p* < 0.05). To assess the impact of residual contact exposure to insecticides on the survival of both parasitoid species and estimate median survival times (LT_50_), time–mortality data were submitted to survival analysis by the Kaplan–Meier log-rank test. Parasitoids alive at the end of the 30-day observations following the insecticide residual contact exposure were treated as censored [54]. Spinosad was excluded from the dataset for *N. tutae* survival analysis because no parasitoid adult survived after 72 h of residual contact exposure to this insecticide (100% adult mortality). In the case of overall log-rank test significance, a paired comparison of any two survival curves was made using the Holm method (*p* < 0.05) (RStudio 2023.12.1).

Data concerning reproductive mortality, non-reproductive mortality, and juvenile survival of *N. tutae*, as well as those related to *G. kimorum* apparent parasitism, were analyzed using the non-parametric Kruskal–Wallis test followed by Dunn’s post-hoc test (*p* < 0.05).

To assess the effect of different treatments (i.e., parasitoid and insecticide alone or their combinations) on the mortality of both pests, the obtained data were previously corrected by means of Abbott’s formula using corresponding control mortalities (i.e., pest exposure to distilled water in absence of parasitism) and analyzed using the Kruskal–Wallis and Dunn’s post-hoc tests (*p* < 0.05). In each treatment, the Mann–Whitney U test (*p* < 0.05) was also used to investigate differences in host pest mortality between the insecticide alone or in combination with the parasitoid. The non-parametric statistical analyses were conducted using IBM^®^ SPSS^®^ Statistics for Macintosh, Version 23.0.0.0 (IBM Corp., Armonk, NY, USA).

## 3. Results

### 3.1. Ganaspis kimorum Mortality during Residual Contact Exposure to Insecticides

Within each treatment, the mortality of *G. kimorum* adults was not significantly different between male and female wasps after 24, 48, or 72 h of residual exposure to the tested insecticides (Mann–Whitney U test: control, 24 h, U = 112.5; *df* = 28; *p* = 1; 48 h, U = 97.5; *df* = 28; *p* = 0.539; 72 h, U = 90; *df* = 28; *p* = 0.367; *B. bassiana*, 24 h, U = 112.5; *df* = 28; *p* = 1; 48 h, U = 112.5; *df* = 28; *p* = 1; 72 h, U = 112.5; *df* = 28; *p* = 1; *B. thuringiensis*, 24 h, U = 105; *df* = 28; *p* = 0.775; 48 h, U = 97.5; *df* = 28; *p* = 0.539; 72 h, U = 105; *df* = 28; *p* = 0.775; cyantraniliprole, 24 h, U = 97.5; *df* = 28; *p* = 0.539; 48 h, U = 97.5; *df* = 28; *p* = 0.539; 72 h, U = 90; *df* = 28; *p* = 0.367; garlic EO, 24 h, U = 105; *df* = 28; *p* = 0.775; 48 h, U = 105; *df* = 28; *p* = 0.775; 72 h, U = 112.5; *df* = 28; *p* = 1; spinosad, 24 h, U = 90; *df* = 28; *p* = 0.367; 48 h, U = 75; *df* = 28; *p* = 0.126; 72 h, U = 90; *df* = 28; *p* = 0.367). Therefore, data on male and female survival were pooled to analyze the differences among insecticides and the pooled results are presented.

**Figure 1:**
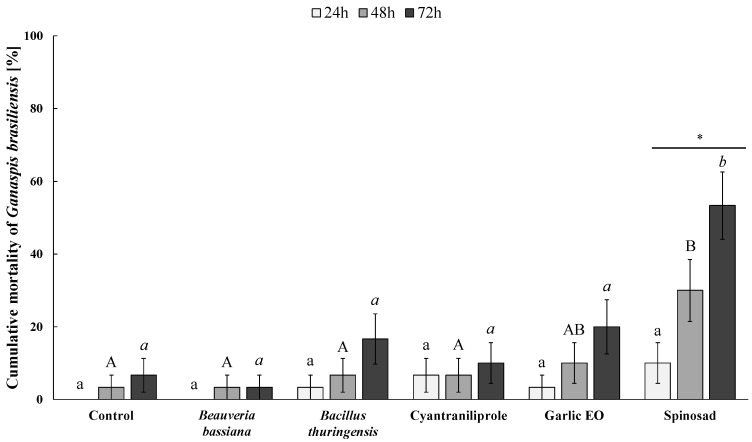
Cumulative mortality (Mean % ± SE) of *Ganaspis kimorum* adults evaluated at 24, 48, and 72 h of exposure to each tested insecticide. Different lower-case letters, capital letters, and lower-case italic letters indicate significant differences among insecticides after 24, 48, and 72 h of exposure, respectively (Kruskal–Wallis H test followed by Dunn’s post-hoc test with Bonferroni-corrected *p*-values, *p* ≤ 0.05). Within each treatment, asterisks show significant differences among the three exposure durations (Kruskal–Wallis H test followed by Dunn’s post-hoc test with Bonferroni-corrected *p*-values, *p* ≤ 0.05).

The mortality of the parasitoid was not significantly different within the first 24 h of exposure to different insecticide residues (*H* = 6.060; *df* = 5; *p* = 0.300) but was significantly affected by the insecticide treatment after 48 (*H* =16.942; *df* = 5; *p* = 0.005) and 72 h (*H* = 33.099; *df* = 5; *p* < 0.001) of insecticide exposure (Figure 1). Among the tested insecticides, only spinosad caused a significant increase in parasitoid mortality over the last two days of exposure. The highest acute toxicity of spinosad was observed following 72 h of exposure (53.3 ± 9.3% mortality), which was not significantly different than the mortality at 48 h (30.0 ± 8.5%) but was significantly higher than that at 24 h (10.0 ± 5.6%) (*H* = 13.02; *df* = 2; *p* = 0.001) (Figure 1). The other treatments did not show significant effects on the parasitoid mortality over the three days of exposure (control, *H* = 2.046; *df* = 2; *p* = 0.360; *B. bassiana*, *H* = 1.011; *df* = 2; *p* = 0.603; *B. thuringiensis*, *H* = 3.527; *df* = 2; *p* = 0.171; cyantraniliprole, *H* = 0.306; *df* = 2; *p* = 0.858; garlic EO, *H* = 4.228; *df* = 2; *p* = 0.121) (Figure 1).

### 3.2. Necremnus tutae Mortality during Residual Contact Exposure to Insecticides

The mortality of *N. tutae* adults was not significantly affected by the sex within the 72 h of residual exposure to each of the tested insecticides (Mann–Whitney U test: control, 24 h, U = 120; *df* = 28; *p* = 0.775; 48 h, U = 127.5; *df* = 28; *p* = 0.539; 72 h, U = 135; *df* = 28; *p* = 0.367; *B. bassiana*, 24, U = 105; *df* = 28; *p* = 0.775; 48 h, U = 120; *df* = 28; *p* = 0.775; 72 h, U = 112.5; *df* = 28; *p* = 1; *B. thuringiensis*, 24 h, U = 112.5; *df* = 28; *p* = 1; 48 and 72 h, U = 120; *df* = 28; *p* = 0.775; chlorantraniliprole, 24 h, U = 105; *df* = 28; *p* = 0.775; 48 h, U = 120; *df* = 28; *p* = 0.775; 72 h, U = 112.5; *df* = 28; *p* = 1; garlic EO, 24 and 48 h, U = 112.5; *df* = 28; *p* = 1; 72 h, U = 90; *df* = 28; *p* = 0.367; spinosad, 24 h, U = 82.5; *df* = 28; *p* = 0.217; 48 and 72 h, U = 112.5; *df* = 28; *p* = 1). Differences among insecticides were thus analyzed by pooling the data on male and female survival. 

**Figure 2:**
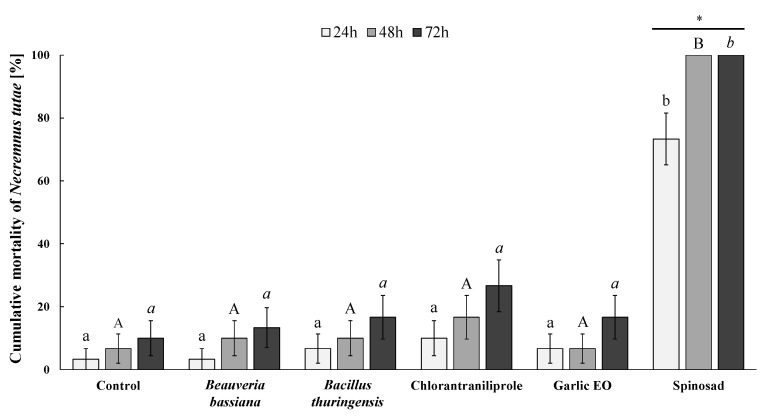
Cumulative mortality (Mean % ± SE) of *Necremnus tutae* adults evaluated at 24, 48, and 72 h of exposure to each tested insecticide. Different lower-case letters, capital letters, and lower-case italic letters indicate significant differences among insecticides after 24, 48, and 72 h of exposure, respectively (Kruskal–Wallis H test followed by Dunn’s post-hoc test with Bonferroni-corrected *p*-values, *p* ≤ 0.05). Within each treatment, asterisks show significant differences among the three exposure durations (Kruskal–Wallis H test followed by Dunn’s post-hoc test with Bonferroni-corrected *p*-values, *p* ≤ 0.05).

Bioassays showed a significant effect of the insecticide treatment on *N. tutae* mortality after 24 h (*H* = 78.715; *df* = 5; *p* < 0.001), 48 h (*H* = 108.461; *df* = 5; *p* < 0.001), or 72 h (*H* = 83.551; *df* = 5; *p* < 0.001) of residual exposure (Figure 2). Spinosad was the most toxic compound and significantly killed more wasps than any other treatment and caused 100% mortality after 48 or 72 h of exposure, which was significantly higher than the mortality during the first day of exposure (73.3 ± 8.2%) (*H*= 17.366; *df* = 2; *p* < 0.001) (Figure 2). In contrast, no significantly different toxicity was found for the other treatments over the three days of exposure (control, *H* = 1.060; *df* = 2; *p* = 0.589; *B. bassiana*, *H* = 1.899; *df* = 2; *p* = 0.387; *B. thuringiensis*, *H* = 1.557; *df* = 2; *p* = 0.459; chlorantraniliprole, *H* = 2.856; *df* = 2; *p* = 0.240; garlic EO, *H* = 2.198; *df* = 2; *p* = 0.333) (Figure 2).

### 3.3. Insecticide Effects on Ganaspis kimorum Parasitism

**Figure 3:**
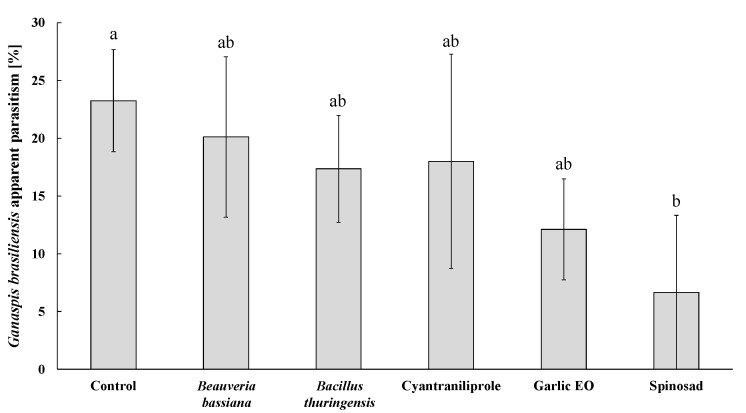
Insecticide effects on the apparent parasitism of *Ganaspis kimorum* females parasitizing *Drosophila suzukii* larvae in a treated blueberry during 72 h of residual contact exposure. Means (±SE) with different letters are significantly different according to Kruskal–Wallis H test followed by Dunn’s post-hoc test for multiple comparisons, at *p* ≤ 0.05.

The apparent parasitism of *G. kimorum* on *D. suzukii* was significantly affected by the insecticide treatment (*H* = 11.09; *df* = 5; *p* = 0.049). Exposure of the parasitoid to untreated blueberries (control) resulted in an apparent parasitism of 23.2 ± 4.4%. Spinosad was the only compound that significantly reduced the parasitism by 3.46-fold when compared to the control treatment, and there was no significant difference among other insecticide treatments (Figure 3).

### 3.4. Insecticide Effects on Necremnus tutae Parasitism and Host Killing

The non-reproductive mortality (host feeding + host killing) caused by *N. tutae* on *T. absoluta* larvae was significantly affected by the treatment (*H* = 56.190; *df* = 5; *p* < 0.001) (Table 1). In particular, it was significantly higher for parasitoids exposed to distilled water (control), *B. thuringiensis*, and *B. bassiana*, when compared to the other three treatments, accounting for a high proportion of total host mortality (Table 1). The percentage of *T. absoluta* larvae killed due to the parasitoid’s non-reproductive activity greatly decreased by 2.76-fold, 6.27-fold, and 17.25-fold for chlorantraniliprole, garlic EO, and spinosad, respectively, compared to the untreated control. Similarly, the proportion of parasitized host larvae (reproductive mortality) significantly differed among treatments (*H* = 41.050; *df* = 5; *p* < 0.001). The highest reduction in parasitized *T. absoluta* larvae occurred when *N. tutae* was exposed to spinosad (11.50-fold) and garlic EO (4.93-fold) (Table 1). The proportion of *N. tutae* eggs developed into adults (parasitoid juvenile survival) was also significantly affected by the treatment (*H* = 23.400; *df* = 5; *p* < 0.001) (Table 1). In particular, chlorantraniliprole and garlic EO reduced *N. tutae* juvenile survival by almost half, while spinosad treatment resulted in no survival of the parasitoid offspring (Table 1).

### 3.5. Ganaspis kimorum Survival after Residual Contact Exposure to Insecticides

**Figure 4:**
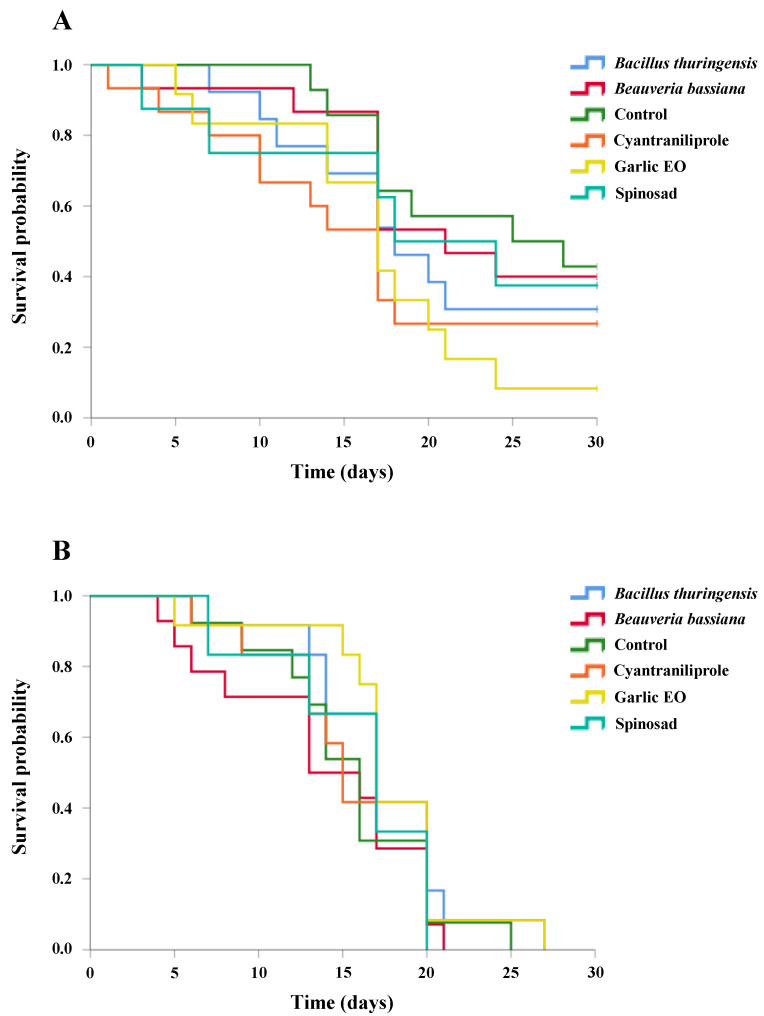
Survival curves of *Ganaspis kimorum* female (**A**) and male (**B**) adults following 72 h of residual contact exposure to different insecticides. The curves were generated through Kaplan–Meier estimators and no significant differences (*p* < 0.05) were obtained when compared in the log-rank test (*p* < 0.05).

No significant differences among treatments were found for the survival rates of both *G. kimorum* males and females after insecticide residual contact exposure (log-rank test: males: *χ*^2^ = 2.990, *df* = 5, *p* = 0.702; females: *χ*^2^ = 5.980, *df* = 5, *p* = 0.308). The lowest LT_50_ values for *G. kimorum* females after insecticide exposure were observed for cyantraniliprole (16.5 ± 2.4 days) and garlic EO (16.9 ± 1.9 days) treatments, while the highest mean survival of parasitoid females was observed for the control group (23.6 ± 1.8 days) (Figure 4A). The estimated LT_50_ values of parasitoid males ranged from 13.8 ± 1.6 days after residual exposure to *B. bassiana* to 17.6 ± 1.5 days after residual exposure to the garlic EO residue, while it was 15.5 ± 1.40 days for parasitoid males from the control group (Figure 4B).

### 3.6. Necremnus tutae Survival after Residual Contact Exposure to Insecticides

**Figure 5:**
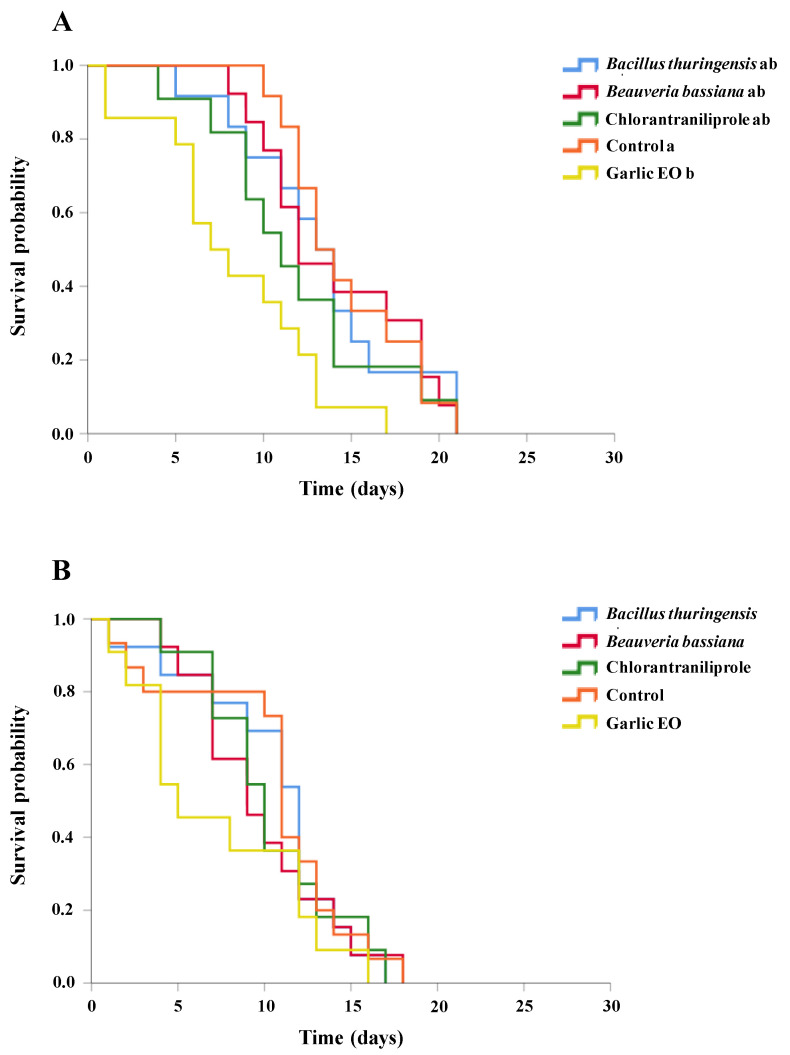
Survival curves of *Necremnus tutae* female (**A**) and male (**B**) adults following 72 h of residual contact exposure to different insecticides. The curves were generated through Kaplan–Meier estimators and compared in the log-rank test (*p* < 0.05). Treatment names with letters identify significantly different curves.

The survival analysis of *N. tutae* females after residual contact exposure to insecticides revealed significant differences between treatments (log-rank test: *χ*^2^ = 14.005, *df* = 4, *p* = 0.007), with only garlic EO significantly reducing the parasitoid’s mean survival time (8.3 ± 1.2 days) compared to that of the control (14.7 ± 1.0 days) (Holm comparison method: *p* = 0.024) (Figure 5A). The mean LT_50_ values for *N. tutae* adult females exposed to the other treatments were not significantly different from the control, ranging from 11.8 ± 1.5 days to 13.2 ± 1.4 days and 14.1 ± 1.3 days, for chlorantraniliprole, *B. thuringiensis*, and *B. bassiana*, respectively. No significant difference was found in the survival rates of *N. tutae* males exposed to different insecticides (log-rank test: *χ*^2^ = 2.302, *df* = 4, *p* = 0.680). However, mean LT_50_ values ranged from 9 to 11 days for all treatments except for garlic EO, which showed the lowest LT_50_ (7.36 ± 1.53 days) (Figure 5B).

### 3.7. Individual and Combined Insecticide-Ganaspis kimorum Impact on Pest Mortality

**Figure 6:**
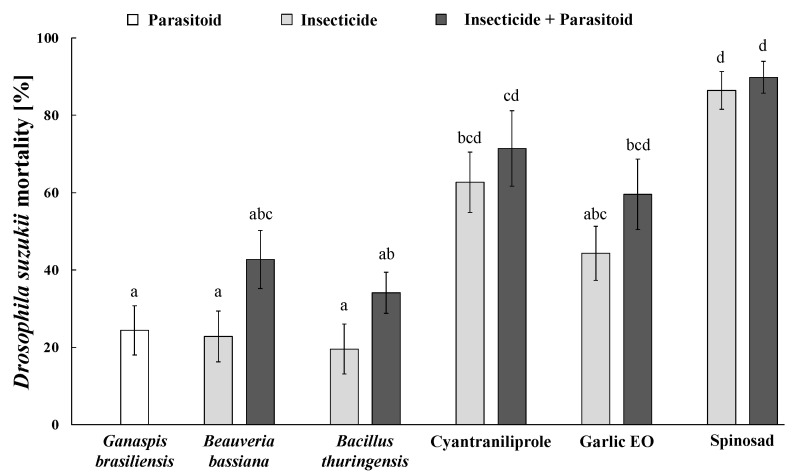
Individual and combined impacts of *Ganaspis kimorum* and insecticides on *Drosophila suzukii* mortality. Data were corrected by means of Abbott’s formula using corresponding control mortalities. Means (±SE) with different letters are significantly different according to Kruskal–Wallis H test followed by Dunn’s post-hoc test for multiple comparisons, at *p* ≤ 0.05. Within each treatment, the impact of the insecticide on *D. suzukii* mortality was statistically similar to that caused by its combination with *G. kimorum* (Mann–Whitney U test, *p* ≤ 0.05).

*Ganaspis kimorum*, tested insecticides, and their combination significantly affected *D. suzukii* mortality (*H* = 74.35; *df* = 10; *p* < 0.001). *Ganaspis kimorum* alone caused 24.4 ± 4.4% of host mortality. For each insecticide, the *D. suzukii* mortality increased in combination with *G. kimorum*, although not significantly according to the Mann–Whitney U test (*B. bassiana*, U = 156.5; *df* = 28; *p* = 0.067; *B. thuringiensis*, U = 154.5; *df* = 28; *p* = 0.081; cyantraniliprole, U = 135; *df* = 28; *p* = 0.367; garlic EO, U = 144.5; *df* = 28; *p* = 0.187; spinosad, U = 122; *df* = 28; *p* = 0.713). However, *G. kimorum* in combination with *B. bassiana* and *B. thuringiensis* increased *D. suzukii* mortality by 1.87 and 1.74 times compared to the microbials alone. Spinosad and cyantraniliprole alone caused higher pest mortalities (86.4 ± 4.9 and 62.7 ± 7.8%, respectively) among the tested insecticides, and in both cases, *G. kimorum* contributed to the lowest host mortalities, equal to 3.9 and 13.9% more *D. suzukii* deaths than individual pesticide applications, respectively (Figure 6).

### 3.8. Individual and Combined Insecticide–Necremnus tutae Impact on Pest Mortality

**Figure 7:**
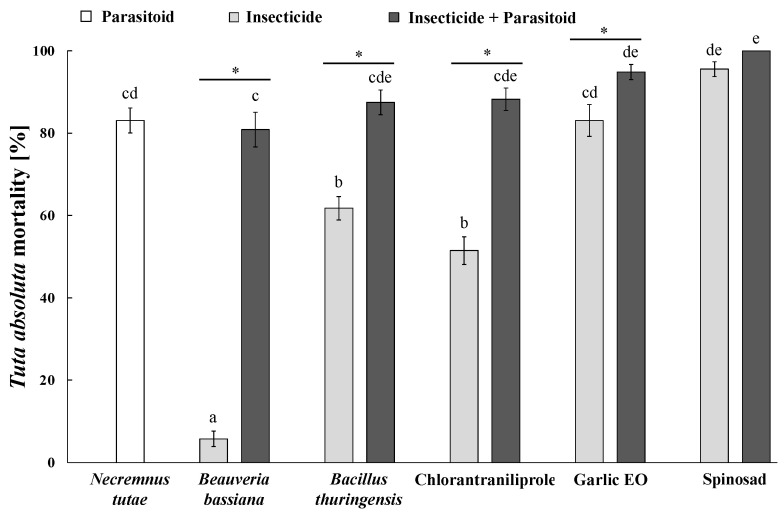
Individual and combined impact of *Necremnus tutae* and insecticides on *Tuta absoluta* mortality. Data were corrected by means of Abbott’s formula using corresponding control mortalities. Means (±SE) with different letters are significantly different according to Kruskal–Wallis H test followed by Dunn’s post-hoc test for multiple comparisons at *p* ≤ 0.05. Within each treatment, asterisks show significant differences in *T. absoluta* mortality between the insecticide alone and its combination with *N. tutae* according to the Mann–Whitney U test, *p* ≤ 0.05.

*Necremnus tutae* alone killed 83.1 ± 3.0% of *T. absoluta* larvae and significantly increased host mortality in combination with tested insecticides (*H* = 108.92; *df* = 10; *p* < 0.001). In particular, *N. tutae* increased the pest mortality by 14.1 times, when compared to the mortality caused by *B. bassiana* alone (U = 225; *df* = 28; *p* < 0.001). Chlorantraniliprole and *B. thuringiensis* alone caused similar mortalities (51.5 ± 3.4 and 61.8 ± 2.8%, respectively), but in combination with the parasitoid, the pest mortalities (88.2 ± 2.7 and 87.5 ± 3.0%, respectively) significantly increased by 1.7 (U = 218; *df* = 28; *p* < 0.001) and 1.4 (U = 202; *df* = 28; *p* < 0.001) times, respectively. Host mortality caused by garlic EO alone was 83.1 ± 3.9%, which significantly increased up to 94.9 ± 1.8% in combination with *N. tutae* (U = 166.5; *df* = 28; *p* < 0.023). In contrast, spinosad was the most toxic compound toward *T. absoluta* larvae (95.6 ± 1.8%), and *N. tutae* did not provide any statistically significant additive impact on pest mortality (U = 122; *df* = 28; *p* = 0.713) (Figure 7).

## 4. Discussion

In this study, we evaluated the impact of four bioinsecticides compared to a synthetic one on two invasive pests, *D. suzukii* and *T. absoluta*, and their major larval parasitoids, *G. kimorum* and *N. tutae*, respectively. To the best of our knowledge, this is the first study investigating the non-target impact and compatibility of *B. thuringiensis*, *B. bassiana,* and garlic EO with both parasitoid species. Moreover, we provide the first data on the lethal and sublethal effects of spinosad and chlorantraniliprole on *N. tutae*. In the current study, the effect of the parasitoids on host mortality results from the survival rate of adult wasps within the 72 h contact exposure to insecticide residues and the sublethal effects of this exposure on their reproductive behaviors. In addition, parasitoid performance will be also affected by the availability of host larvae following insecticide treatment. In the longer term, the survival of juvenile parasitoids will also have an impact on natural enemy populations. Overall, our results revealed different compatibilities of the tested insecticides with both parasitoids in terms of the non-target effects on the parasitoids and their combined effects on host mortality. 

Insecticides alone significantly reduced *D. suzukii* larvae survival. We found that the combinations of tested insecticides with *G. kimorum* increased the overall host mortalities by 3.4% to 19.9%, but this result was not statistically significant. The parasitoid contributed more to *D. suzukii* mortality in combination with the insecticides (both tested microbials and the garlic EO) that had no or the least toxicity to adult wasps during the 72 h of residual exposure. The low contribution by *G. kimorum* may be due to the low parasitism (24.4%) even on untreated blueberries. In contrast, *N. tutae* significantly increased the total host mortality in combination with most of the tested insecticides, except for spinosad, which killed all adult wasps after 48 h of exposure. 

*Necremnus tutae* was very effective on *T. absoluta,* parasitizing and/or killing a high proportion of host larvae on untreated host leaves, causing approximately 82% of pest mortality. The parasitoid killed more hosts by host-feeding or host-killing activity than parasitism when it was tested alone (control group) (Table 1). These results corroborate those reported in previous studies on *N. tutae* behavioral shift and life history traits and also indicated a higher proportion of killed (non-reproductive effects) than parasitized (reproductive effects) *T. absoluta* host larvae [47,55,56]. 

The two entomopathogens (*B. bassiana* and *B. thuringiensis*) can be considered safe and compatible with both *G. kimorum* and *N. tutae* due to their lack of non-target effects and higher host mortality observed in all microbial–parasitoid combinations compared to the scenario in which they are used alone. Indeed, *N. tutae* combined with *B. bassiana* and *B. thuringiensis* increased *T. absoluta* mortality by 75.1% and 25.7% (Figure 7), respectively, while *G. kimorum* increased *D. suzukii* mortality by 19.9% and 14.6%, respectively, although not significantly (Figure 6). Indeed, both *B. bassiana* and *B. thuringiensis* are characterized by poor or low penetration rates into fruits and may have no or minimal toxicity toward immature *D. suzukii* [57] and developing parasitoid offspring in the host larvae. Indeed, this study showed no effects of the two microbials on the offspring survival of *G. kimorum* (Figure 3). However, *B. thuringiensis* was found to impair the fly larval endoparasitoid *Leptopilina* spp. (Hymenoptera: Figitidae) developing in contaminated *Drosophila melanogaster* Meigen (Diptera: Drosophilidae) larvae, which typically breed on rotten fruits that could be susceptible to insecticide spray drifts [58]. This situation would be unlikely to occur for *G. kimorum* because it parasitizes only *D. suzukii* larvae developing within fresh fruits on the plant canopy [51]. Because *B. bassiana* exhibits endophytic and epiphytic activity against *T. absoluta* in tomato crops [59] and *B. thuringiensis* targets moth larvae, non-target effects would be expected on the survival of immature parasitoids indirectly through affected host larvae. However, no harmful effect of both microbials was found on *N. tutae* juvenile survival in this study (Table 1).

The selectivity of the two microbial insecticides strongly depends on their main mechanisms of action. Indeed, *B. bassiana* infects insects percutaneously and conidia germination is typically completed around 20 h after contact. This process is strongly influenced by insect cuticular lipids, which possess antimicrobial properties affecting the attachment of the spores to the cuticle and their germination and penetration into the insect body, where the fungus will also need to overcome the host immune defenses [60]. Therefore, *B. bassiana* was reported to have a minimal or absent impact on non-target organisms [61], especially on adult parasitoids [60]. In addition, the host-specificity of *B. bassiana* is a strain-specific trait, and insects that are infected in the laboratory may not necessarily be infected in nature or under field conditions [60]. The effect of *B. thuringiensis* on natural enemies has been proven to be minimal because of the inadequate midgut environment for successful intoxication [62]. In support of these considerations, different studies showed that the integration of *B. thuringiensis* with *Trichogramma* spp. egg parasitoids (Hymenoptera: Trichogrammatidae) significantly decreased *T. absoluta* infestations in field conditions [63,64]. 

Garlic EO was safe for *G. kimorum* but caused sublethal toxicity to *N. tutae*. Garlic EO is a promising botanical compound against both *T. absoluta* and *D. suzukii* [29,65]. The current study showed the compatibility of garlic EO with *G. kimorum* for the first time, as well as the sublethal toxicity of *N. tutae*. This botanical insecticide impaired adult *N. tutae* reproductive and non-reproductive behaviors, as well as the survival of immature (Table 1) and adult female wasps following residual exposure (Figure 5). Similarly, Passos et al. [39] and Ricupero et al. [29] found that garlic EO inhibited the behavior and fertility of a key predator of *T. absoluta*, i.e., *Nesidiocoris tenuis* (Reuter) (Hemiptera: Miridae). However, more studies are needed to draw definitive conclusions on the impact of garlic EO on parasitoids. Giunti et al. [35] recently reviewed the non-target impact of essential oil-based biopesticides on natural enemies and showed that their toxicity assessment needs multiple factors including the plant origin, application methods, host and parasitoid species, and stages, as they can affect the results of toxicity assessment of oil-based biopesticides. 

In contrast to *B. bassiana* and *B. thuringiensis* (which are commonly used) and garlic EO (which is under evaluation for use in the tomato and soft-skinned fruit agroecosystems against other pests), cyantraniliprole and chlorantraniliprole are commonly used synthetic insecticides for the conventional management of *D. suzukii* and *T. absoluta*, respectively [6,10]. Therefore, the impacts of these two synthetic insecticides on *G. kimorum* and *N. tutae* would be highly expected in the field. Cyantraniliprole affected *D. suzukii* larvae in the fruit [66,67,68] and the key physiological traits of *D. suzukii* larvae at sublethal doses in the rearing diet [28]. It is, thus, very effective for the control of *D. suzukii* but has been considered a low-risk insecticide for non-target species [69]. The current study also showed that cyantraniliprole was effective (alone killed 62.7% of *D. suzukii* larvae) and had no effect on *G. kimorum*. Our complementary study indicated that cyantraniliprole LC_50_ on *G. kimorum* was higher than the recommended field dose against *D. suzukii* and that this synthetic insecticide did not impair the parasitoid survival [70].

Chlorantraniliprole was widely reported to be highly effective against *T. absoluta* larvae [6]. The 51.8% of host mortality found in the current study using the recommended label rate could be related to the widespread resistance of this pest to chlorantraniliprole across the Mediterranean region [6], including Italy where the pest colony used in this study was initiated. Although chlorantraniliprole is generally considered harmless to natural enemies [71,72], several studies also showed negative effects on the parasitoid’s reproduction and offspring emergence [73]. In this study, chlorantraniliprole was also found to moderately impair the reproductive and non-reproductive behavioral traits of *N. tutae*, as well as its offspring survival (Table 1). Although the combination of chlorantraniliprole with *N. tutae* determined a significantly higher host mortality (88.2%) than the insecticide alone (Figure 7), it is not recommended for the combination use of this insecticide with the parasitoid. Still, chlorantraniliprole can be used as part of IPM approaches as this insecticide could also target other key pests in a given crop.

Lastly, spinosad was the most effective insecticide against *T. absoluta* and *D. suzukii* but also had the most detrimental effect on *N. tutae* and *G. kimorum*. As a consequence, both parasitoids contributed least to their host mortality (4.4% and 2.9%, respectively) in combination with spinosad. Spinosad can be absorbed into the fruit tissues through the oviposition holes made by flies [74]. The mortality of immature *D. suzukii* ranged from 80-90% under laboratory bioassays (e.g., fruit dipping in insecticide solution) [67,68] and field spray applications [66,75]. This study showed that spinosad alone killed 86.4% of *D. suzukii* in the fruits (Figure 6). Spinosad is one of the main tools for the conventional and organic management of *T. absoluta* due to its high effectiveness in killing larvae in the leaf mines [6,38]. It alone killed 95.6% of *T. absoluta* larvae in the present study (Figure 7).

Spinosad has been classified as moderately harmful or harmful (IOBC classes 3 and 4, respectively) to hymenopteran parasitoids, and thus is questionable regarding its potential compatibility with key biological control agents [34]. High acute toxicity of spinosad at label field rates was reported on more than 20 parasitoid species more than a decade ago [34]. Not surprisingly, spinosad was found to impair the survival of juvenile and adult *G. kimorum* in this study (Figure 1 and Figure 3). Even doses more than 10,000 times lower than the label rate against *D. suzukii* were found to affect this parasitoid’s life-history traits [70]. Thus, spinosad should not be considered compatible with *G. kimorum*, as it is with other key *D. suzukii* parasitoids, such as *Pachycrepoideus vindemiae* (Rondani) (Hymenoptera: Pteromalidae), *Trichopria drosophilae* (Perkins), and *T. anastrephae* Lima (Hymenoptera: Diapriidae) [28,36,76]. Spinosad was extremely toxic to *N. tutae*, resulting in 100% adult mortality after 48 h of exposure to treated leaves, and significantly impaired the parasitoid’s reproductive ability and no offspring could survive (Table 1). Other studies also showed that spinosad impaired the effectiveness of *T. absoluta* parasitoids [77,78], particularly that of the larval ectoparasitoid *Bracon nigricans* Szépligeti (Hymenoptera: Braconidae), which is among the major natural enemies of *T. absoluta* in the Mediterranean area [37,79], together with *N. tutae* [48]. However, some studies suggested that parasitoid releases combined with this bioinsecticide have the potential to control *T. absoluta* infestations in tomato crops in the fields [38,80]. Further studies are needed to confirm the impact of this insecticide, as well as of the other tested bioinsecticides, on *N. tutae* under field conditions.

## 5. Conclusions

*Ganaspis kimorum* did not significantly increase host mortality in combination with tested insecticides and this was likely due to the low parasitism under the current experimental setting, while *N. tutae* significantly showed an additive effect on host mortality. The two tested entomopathogens can be considered compatible with both parasitoids, and in particular, *B. thuringiensis* is the most promising biocontrol agent for use in combination with *N. tutae* due to its importance for the organic management of *T. absoluta*. Garlic EO is a promising insecticide for controlling both pests, but its use is recommended mainly with *G. kimorum* because of its non-target effect on *N. tutae*, as also observed for chlorantraniliprole. Moreover, cyantraniliprole is currently one of the main options for the conventional management of *D. suzukii,* and our results suggest its suitability for integration with *G. kimorum* because it did not affect the parasitoid’s fitness but provided successful control of *D. suzukii* larvae in the fruits. Although spinosad is a mainstay of the conventional and organic management of both *D. suzukii* and *T. absoluta*, the origin of a given compound does not guarantee its safety and its combination with *G. kimorum* and *N. tutae* should be avoided due to the impactful non-target toxicity on both parasitoids. These results provide practical insights into biological control-centered IPM packages targeting these two major invasive pests worldwide.

## Figures and Tables

**Table 1:** Results (mean % ± SE) of the assessment of the insecticide impact on the host–parasitoid interactions after 72 h of residual contact exposure to treated tomato leaves. Reproductive mortality shows the proportion of parasitized hosts (*T. absoluta* larvae) with *N. tutae* eggs in the mines over the total number of exposed hosts. Non-reproductive mortality identifies the proportion of hosts who died of host-feeding and host-killing by *N. tutae* over the total number of exposed hosts. Parasitoid juvenile survival shows the proportion of *N. tutae* adults successfully emerged over the total number of parasitized *T. absoluta* larvae (reproductive mortality). Within each column, different letters indicate significant differences among treatments (Kruskal–Wallis test followed by Mann–Whitney pairwise post-hoc test, *p* ≤ 0.05).

Treatment	Non-Reproductive Mortality	Reproductive Mortality	Parasitoid Juvenile Survival
Control (water)	46.00 ± 3.06 a	36.00 ± 3.88 a	60.89 ± 7.79 a
*Beauveria bassiana*	36.00 ± 3.49 a	34.00 ± 3.75 ab	61.56 ± 7.89 a
*Bacillus thuringiensis*	38.67 ± 3.76 a	29.33 ± 4.52 ab	42.56 ± 9.76 ab
Chlorantraniliprole	16.67 ± 3.98 b	23.33 ± 3.86 b	36.67 ± 8.94 b
Garlic EO	7.33 ± 2.48 bc	9.33 ± 2.67 c	36.67 ± 11.52 b
Spinosad	2.67 ± 1.18 c	4.00 ± 1.90 c	0.00 c

## Data Availability

The data presented in this study are available upon request from the corresponding author.
